# Changes of Physical Activity and Ultra-Processed Food Consumption in Adolescents from Different Countries during Covid-19 Pandemic: An Observational Study

**DOI:** 10.3390/nu12082289

**Published:** 2020-07-30

**Authors:** María Belén Ruíz-Roso, Patricia de Carvalho Padilha, Diana C. Matilla-Escalante, Paola Brun, Natalia Ulloa, Diofanor Acevedo-Correa, Wilza Arantes Ferreira Peres, Miquel Martorell, Thais Rangel Bousquet Carrilho, Letícia de Oliveira Cardoso, Fernanda Carrasco-Marín, Katherine Paternina-Sierra, María-Carmen Lopez de las Hazas, Jhon E. Rodriguez-Meza, Luisa F. Villalba-Montero, Giulia Bernabè, Anthony Pauletto, Xhoajda Taci, Richar Cárcamo-Regla, J. Alfredo Martínez, Alberto Dávalos

**Affiliations:** 1 Laboratory of Epigenetics of Lipid Metabolism, Madrid Institute for Advanced Studies (IMDEA)–Food, CEI UAM + CSIC, 28049 Madrid, Spain; diana.mantilla@imdea.org (D.C.M.-E.); mcarmen.lopez@imdea.org (M.-C.L.d.l.H.); 2 Instituto de Nutrição Josué de Castro, Universidade Federal do Rio de Janeiro, Rio de Janeiro 21941-902, Brazil; wilza@nutricao.ufrj.br (W.A.F.P.); thaisrangelnut@gmail.com (T.R.B.C.); 3 Instituto de Puericultura e Pediatria Martagão Gesteira, Universidade Federal do Rio de Janeiro, Rio de Janeiro 21941-902, Brazil; 4 Department of Molecular Medicine, University of Padova, I-35100 Padua, Italy; paola.brun.1@unipd.it (P.B.); giulia.bernabe@student.unife.it (G.B.); anthony.pauletto@phd.unipd.it (A.P.); xhoajda.taci@studenti.unipd.it (X.T.); 5 Centro Vida Saludable, Universidad de Concepción, Concepción 4070386, BíoBío, Chile; nulloa@udec.cl (N.U.); mmartorell@udec.cl (M.M.); fercarrasco@udec.cl (F.C.-M.); ricarcamo@udec.cl (R.C.-R.); 6 Departamento de Bioquímica Clínica e Inmunología, Facultad de Farmacia, Universidad de Concepción, Concepción 4070386, Chile; 7 Research Group in Innovation and Agricultural and Agroindustrial Development, University of Cartagena, Cartagena de Indias 48-152, Colombia; dacevedoc1@unicartagena.edu.co (D.A.-C.); paternina185@gmail.com (K.P.-S.); jrodriguezm3@unicartagena.edu.co (J.E.R.-M.); 8 Departamento de Nutrición y Dietética, Facultad de Farmacia, Universidad de Concepción, Concepción 4070386, Chile; 9 Oswaldo Cruz Foundation, National School of Public Health, Rio de Janeiro 21041-210, Brazil; leticiadeoliveiracardoso@gmail.com; 10 Facultad de Medicina, University of Cartagena, Cartagena de Indias 29-5050, Colombia; lvillalbam1@unicartagena.edu.co; 11 Ciber de la Fisiopatología de la Obesidad y Nutrición (CIBEROBN), Instituto de Salud Carlos III, 28049 Madrid, Spain; jalfredo.martinez@imdea.org; 12 Department of Nutrition and Physiology, Center for Nutrition Research, University of Navarra, IDISNA Navarra, 31008 Pamplona, Spain; 13 Cardiometabolic Nutrition Group, Madrid Institute for Advanced Studies Food (IMDEA Food), CEI UAM + CSIC, 28049 Madrid, Spain

**Keywords:** adolescent, physical activity, Covid-19 pandemic, e-survey, Google Forms

## Abstract

Aim: to describe physical activity and ultra-processed foods consumption, their changes and sociodemographic predictors among adolescents from countries in Europe (Italy and Spain) and Latin America (Brazil, Chile, and Colombia) during the SARS-CoV-2-pandemic period. Methods: Cross-sectional study via web survey. International Physical Activity Questionnaire (IPAQ) and weekly ultra-processed food consumption data were used. To compare the frequencies of physical activity status with sociodemographic variables, a multinomial logistic and a multiple logistic regression for habitual ultra-processed foods was performed. In final models, *p* < 0.05 was considered significant. Results: Sample of 726 adolescents, mostly females (59.6%) aged 16–19 years old (54.3%). Adolescents from Latin America presented odds ratio (OR) 2.98 (CI 95% 1.80–4.94) of being inactive and those whose mothers had higher level of education were less active during lockdown [OR 0.40 (CI 95% 0.20–0.84)]. The habitual ultra-processed consumption was also high during this period in all countries, and more prevalent in Latin America. Conclusion: A higher prevalence of inactivity was observed in this population, but reductions of physical activity and habitual ultra-processed consumption during the pandemic were more pronounced in Latin America. Our findings reinforce the importance of promoting a healthy lifestyle, i.e., exercise and diet, during periods of social isolation.

## 1. Introduction

SARS-CoV-2 caused disruption of daily activities, due to the need for social isolation to slow the progress of the disease. Lockdowns, one of the main measures to curb the spreading of the virus, were promoted by countries in all continents [1]. In addition, the World Health Organization (WHO) advises that a healthy lifestyle can help in the prevention and treatment of the disease [2]. During lockdowns, many countries have developed initiatives to avoid agglomerations, which have led to changes in lifestyle habits, especially those involving food consumption and physical exercise [3].

Adolescence is a crucial stage of human development, when several psychological and social changes occur, in addition to the acquisition of new life habits that are determinants of the health status in adulthood [4]. Among these habits, the practice of physical activity is one of the most important ones. Yet, physical inactivity is common among adolescents and sedentary behaviors in this stage of life have been negatively associated with physical, mental, and social health adverse outcomes [5]. Currently, the WHO estimates that 3 out of 4 adolescents do not meet the minimum recommendations for physical activity [6].

This period of adolescence is characterized by a dynamic development in which the interaction with the social environment shapes the capabilities the individual takes forward into adult life. During adolescence, an individual acquires physical, cognitive, emotional, social, and economic resources that are the foundation for later life health and wellbeing. These same resources define trajectories into the next generation. Investments in adolescent health and wellbeing bring benefits today, for decades to come, and for the next generation [4].

A previous study that described the levels of physical activity in 122 countries, with a total population representing 88.9% of the world population, reported that four-fifths of adolescents do not reach public health guidelines for the recommended levels of physical activity [7].

It is known that active behaviors have been replaced by more sedentary habits [8]. This change has been observed in all socioeconomic levels in several low, middle-, and high-income countries. Data on Brazilian children show that they spend an average of 5 h watching television and increasing time involved with electronic games [8].

Adequate nutrition is also considered a potential factor for health in the early stages of life and adolescence. At this stage, it is important to stimulate good eating behaviors that can both influence the current health status and the predisposition to non-communicable (NCDs) diseases in adulthood. WHO recommends the implementation and maintenance of systems to monitor health risk factors in adolescents [9]. Confinement can exacerbate changes in some lifestyle routines, especially those involving sedentary and eating habits. There is scientific evidence showing that the substitution of homemade and fresh foods with the so-called ultra-processed ones contributes to the increase in the prevalence of overweight, chronic non-communicable diseases, and specific nutritional deficiencies in all life-cycle phases, especially in adolescence [9]. These changes have been observed in all socioeconomic levels worldwide. Hence, considering the current period, in which the population is in social isolation, the need for investigations on the influence of diet quality emerges.

Ammar et al. [10] suggested that the quarantine itself can be considered a risk factor for the consumption of poor-quality foods, such as ultra-processed foods, when compared to the standard living condition. Combined with the potential for lower levels of PA, impaired nutritional habits could lead to a positive energy balance (i.e., weight gain). There is limited evidence to evaluate the effect of confinement on PA and dietary behaviors, especially in adolescence. Investigating how PA and eating behaviors are affected by lengthy restrictions is important to establish appropriate recommendations for lifestyle modifications during this time.

Considering the current scenario of the SARS-CoV-2 pandemic and the measures of social isolation taken by several countries, there is a need to investigate how physical activity practices or sedentary behaviors changed among adolescents and how ultra-processed food consumption is during lockdown, because they represent potential risks factors for future NCDs. So, the aim of our study was to describe physical activity and its changes and to describe ultra-processed foods consumption and its predictors among adolescents from countries in Europe (Italy and Spain) and Latin America (Brazil, Chile, and Colombia) during the SARS-CoV-2-pandemic period.

## 2. Materials and Methods

### 2.1. Study Design

This is a quantitative cross-sectional study, with convenience sampling, consisting of an anonymous electronic survey, also known as an e-survey or web survey. The study was based on a previous validated questionnaire for health promotion evaluation in adolescence [11]. All questions were presented in a differential format, to be answered directly in sequence regarding “before” and “during” confinement conditions, and the participants were guided to compare the situations.

### 2.2. Population, Eligibility Criteria

The study population comprised adolescents residing in Brazil, Chile, Colombia, Spain, and Italy, who filled out the questionnaire. To be included in the research, the adolescent had to be aged between 10 and 19 years and 11 months; had no food restrictions; and provided an authorization to participate in the study, a signed Informed Consent Form, and the Free and Informed Consent Form digitally signed by one of his/her guardians, when necessary.

### 2.3. Data Privacity

During the informed consent process, research participants were confident that all data would be used for research purposes only. Participants’ responses were anonymous and confidential, in accordance with Google’s privacy policy (https://policies.google.com/privacy?hl=en). Participants were not allowed to provide any information to contact information. In addition, participants were able to stop participating in the study and leave the questionnaire at any stage before the end of the process. If they decided to leave the study, their answers would not be saved. The answers were saved just by clicking on the “send” button provided. Upon completing the survey, participants acknowledged their voluntary consent to participate in this anonymous study. Participants were asked to be honest in their responses [10].

### 2.4. Data Collection

Data collection was carried out through a structured questionnaire created in Google Forms (Google LLC, Menlo Park, CA, USA). The questionnaire was self-administered and divided into modules: sociodemographic characteristics, dietary, and lifestyle practices. The survey was administered in Portuguese in Brazil, in Spanish in Spain, Chile, and Colombia and in Italian in Italy. The survey in one of the official languages used (i.e., Spanish) is depicted in Supplementary Table S1. The invitation to participate in the survey was made via social media (Facebook, Instagram, and Whatsapp) and the data collection occurred between 17 April and 20 May.

The periods of lockdown varied according to the evaluated countries, but they all occurred in March. It was considered lockdown in Italy on 9 March 2020; in Spain, 14 March; in Colombia, 24 March, and in Brazil, 27 March 2020. No national lockdown has been established in Chile, but some communities and urban areas did declare a mandatory quarantine at different times. However, on 16 March 2020, schools were closed in that country.

To assess the level of physical activity, the International Physical Activity Questionnaire (IPAQ) was used [12]. This instrument allows classifying the level of physical activity by defining as “active” an adolescent who performs three hundred minutes a week or more of physical activity, in all of the following domains: physical education classes, commuting to school (round trip), physical activity outside and inside the school (extra activities, free time, others).

To compare the status of physical activities before and during social isolation, we created two variables: a complete variable describing the status before/during isolation, with four categories (active before/during; inactive before/active during; inactive before/during; active before/inactive during)—which was one of the main outcomes of this study; a binary variable indicating whether the physical activity status has changed during social isolation (yes/no)—for description purposes only.

For ultra-processed food consumption, we considered the assessment of the weekly consumption of ultra-processed foods, so named according to the NOVA classification [13] based on the question: “In the 7 days, on how many days did you eat...?”, For each of the following foods/food groups: sweets (candies, chocolates, chewing gum, chocolates, or lollipops); soft drinks; and salted industrialized/ultra-processed foods, such as hamburger, ham, bologna, salami, sausage, sausage, instant noodles, packaged snacks, savory biscuits.

Sociodemographic variables were also collected and categorized as follows: age—10–15 years and 16–19 years old; sex—female and male; number of people living in the same home—1–3 people and 4 or more; and maternal education—middle school or less; high school and college.

### 2.5. Data Processing and Statistical Analysis

The use of Google Forms allowed us to work with a dataset in a spread sheet right after data collection. The possibility of restricting answers and options reduced the need to review the dataset. Thus, right after collection, a descriptive analysis of the sample was performed. Absolute and relative frequencies are presented for all variables of interest. Then, to compare the frequency of physical activity status among the categories of the selected variables, Chi-squared tests were performed. Based on the results of these tests, variables with *p* < 0.10 were included in a multinomial logistic regression model. In the final model, all associations with *p* < 0.05 were considered statistically significant, and the odds ratio (OR) and their respective 95% confidence intervals were estimated.

Univariate logistic regression was used to test the independent variables related to the outcome consumption of ultra-processed foods, with *p* < 0.10. Based on the results of this analysis, a multiple regression model was developed using habitual consumption of ultra-processed foods as the dependent variable and the variables that demonstrated a significant association in the initial analysis (*p* < 0.20) as independent variables. In the final model, all associations with *p* < 0.05 were considered significant and their respective 95% confidence intervals were estimated. Data were analyzed in SPSS, version 24.0 [14] and R, version 3.6 [15].

### 2.6. Ethical Issues

The study was approved by the Research Ethics Committee of all the involved countries. The study was registered at the ISRCTN registry (nº. 14025343). The Ethics Committees of the following institutions approved the study: “Comité Ética de Investigación”, Fundación IMDEA Alimentación (IMD PI-043) for Spain; “UFRJ—Instituto de Puericultura e Pediatria Martagão Gesteira of Federal University of Rio de Janeiro” (CAAE 30783320.7.0000.5264) for Brazil; “Comité de Ética, Bioética y Bioseguridad de la Dirección de Investigación y Creación Artística de la Vicerrectoría de Investigación y Desarrollo de la Universidad de Concepción” for Chile; Comité de Ética en Investigaciones de la Universidad de Cartagena” (Acta nº 134) for Colombia; and Università Degli Studi di Padova (33035 22 04 2020) for Italy. The study is in accordance with the ethical principles of non-maleficence, beneficence, justice, and autonomy, contained in the ethical resolutions of each country, according to Helsinki declaration. Informed Consent Form and the Free and Informed Consent Form were signed digitally by one of their guardians before initiating the survey.

## 3. Results

### Socio-Demographic Characteristics and Physical Activity

A total of 734 adolescents initially met the eligibility criteria. Among those, eight (1.1%) declined to participate. The final sample consisted of 726 adolescents; 115 (15.8%) were from Brazil, 170 (23.4%) from Chile, 161 (22.2%) from Colombia, 147 (20.2%) from Spain, and 133 (18.4%) from Italy. Most participants were female (59.6%) and aged 16–19 years (54.3%). In Brazil and Chile, the sample was mostly composed by adolescents aged 10–15 years (58.3% and 62.4%, respectively). In most countries, mothers presented a high level of education, except in Colombia, where most mothers (%) presented lower level. In addition, in all countries, the adolescents presented families with four or more people living in the same house.

The proportion of adolescents considered physically inactive was 73.0% before social isolation and 79.5% during this period. Brazil and Chile were the countries with the highest frequencies of inactive adolescents during isolation. In Brazil, inactivity increased from 40.9% before to 93% during the evaluated period (Table 1).

**Figure 1:**
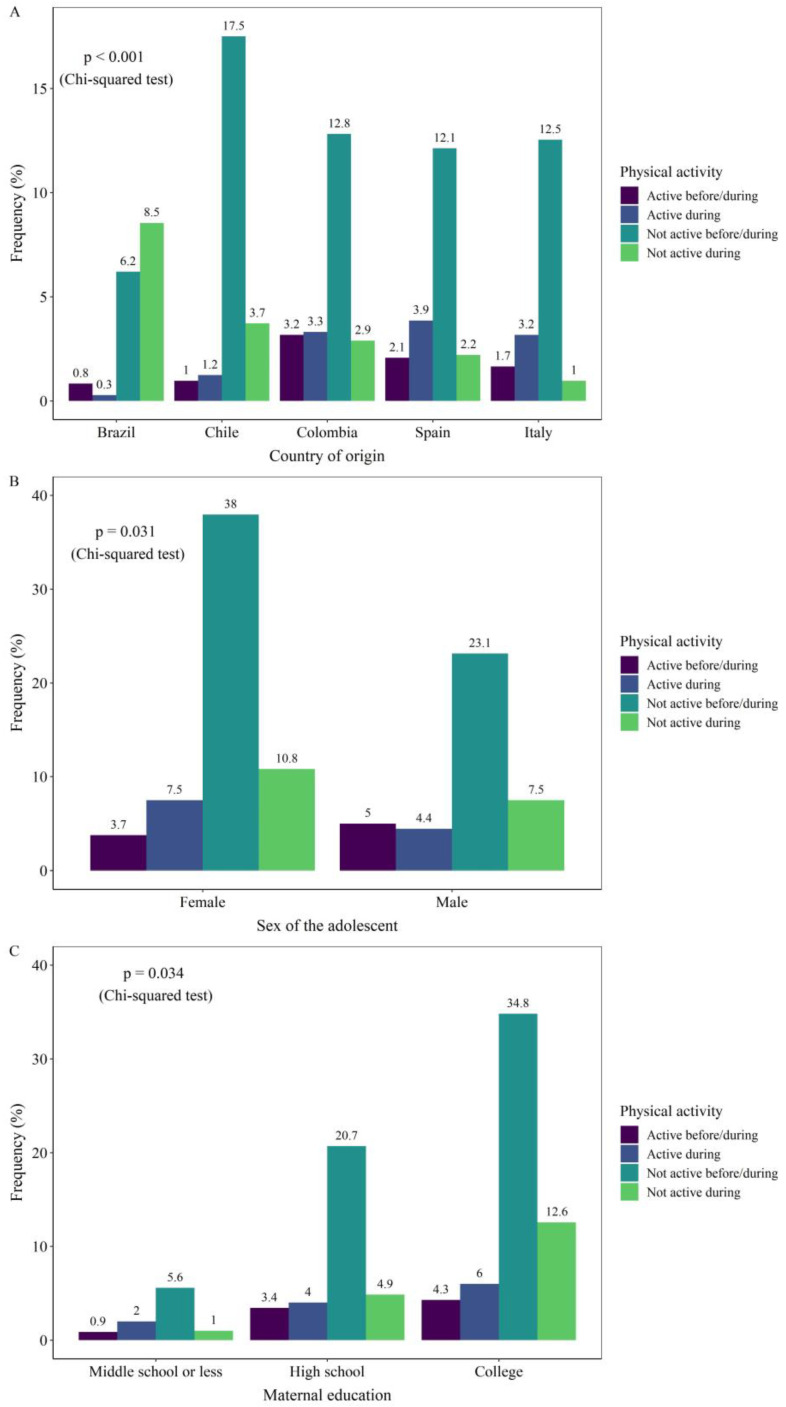
Prevalence of physical activity status before and during social isolation according to (**A**) country; (**B**) Sex of the adolescent; and (**C**) Maternal education.

Figure 1 shows the characteristics of the sample according to the level of physical activity. When the physical activity status before/during social isolation is compared across several sociodemographic characteristics, it is possible to observe a difference in the frequencies according to the country, with Brazil being the country with the highest prevalence of physical inactivity before/after social isolation (*p* < 0.001). The frequency was also different according to continent (*p* = 0.001), sex (*p* = 0.031), number of people living in the same house (*p* = 0.001), and maternal education (*p* = 0.034; Table 2).

The final multivariate model was adjusted by continent, sex, number of people living in the same house, and maternal education (Table 3). It is shown that, after adjustment, only maternal education, sex, and continent seemed to play a role in the odds of physical activity/inactivity during the evaluated period. Adolescents living in Latin America presented an odds ratio (OR) of 2.98 (CI 95% 1.80–4.94) of being physically inactive during quarantine. On the other hand, boys were more active [OR 2.22 (CI 95% 1.28–3.86)] before/during quarantine. Adolescents whose mothers presented high level of education were less active during lockdown [OR 0.40 (CI 95% 0.20–0.84)].

Table 4 presents the multiple regression model for ultra-processed foods consumption. It is observed that living in Latin America (OR 1.58; *p* = 0.007) was associated with habitual ultra-processed foods consumption.

## 4. Discussion

We observed a high prevalence of physical inactivity among adolescents, before and during quarantine, regardless of the country. However, we identified differences in adolescents’ physical activity, especially in the change of its status during COVID-19 lockdown according to the evaluated country. The consumption of ultra-processed food was also high during this period in all countries, but their habitual use was more prevalent in Latin America.

Being physically active benefits mental, physical, and social health [5,16], yet, existing evidence suggests a global pandemic of physical inactivity [4]. Abreu et al. [17], in a study evaluating the reasons for medical consultations by adolescents, identified that most of the diseases diagnosed in this population have a strong behavioral and social component, with emphasis on mental illness and obesity. Such findings highlight the need for a global approach to adolescents, valuing healthy lifestyle practices [6].

A high percentage of school going adolescents are estimated to be insufficiently physically active worldwide [6], with a significant variation between countries [4,5]. Documenting and understanding these differences are important to identify countries and corresponding policies associated with the determinants of physical activity levels [5].

There is evidence that physical activity levels among adolescents are particularly high in European countries [18,19]. Previous cross-country comparisons of adolescent physical activity [5,16] have so far produced limited evidence for a number of reasons. In our survey, the Latin American countries (Brazil, Chile, and Colombia) presented lower levels of physical activity before and during quarantine, showing that inactivity was already a problem, possibly aggravated by social isolation and reduced urban mobility. Our data corroborate those from a meta-analysis with a sample from 52 countries, demonstrating the higher prevalence of physical inactivity among adolescents in Latin America, i.e., Brazil, Chile, and Colombia [5].

The cross-country differences in physical activity levels are probably affected by determinants operating through several levels of influence, such as individual, social, environmental, and political aspects. These include economic factors, which partly determine the resources and the quality of the environments that facilitate participation, including the opportunities available for active transportation to and from school; and cultural factors, such as country-level beliefs regarding the importance of physical activity for health and individual or group identity [5].

Factors such as country economic development and income inequality are noted in highly cited studies as being determinants of adolescent health [20,21]. Yet, previous studies have inconsistently associated such determinants with cross-national differences in adolescent physical activity levels [5]. Our findings add to this evidence, as we observed that national levels of income inequality strongly negatively correlated with levels of activity. This underscores the need to study whether national levels of income inequality have direct effects on activity participation and what other factors cause this effect, e.g., economic development, changing patterns of transportation, increased use of technology and urbanization [5], which might concomitantly contribute to the observed associations [19,22]. Regarding gender differences, many studies identify boys as being more active than girls [18,19]. Cultural factors in general are difficult to evaluate, but may be potentially fruitful targets for identification and modification to increase activity levels. This may also partly explain the gender disparities in physical activity, with boys being more active than girls. However, specific factors explain these disparities are still to be fully elucidated [20].

The relationship between household income and child physical activity shows high between-country variation, with a positive correlation observed in high-income countries and a negative one usually observed in lower-income regions [5,18]. Similar interactions have been reported for childhood obesity and physical activity levels in adults [16]. These findings are consistent with epidemiological observations of nutrition and physical activity changes occurring during the last years. Furthermore, country level factors, such as per-capita income and income inequality are diversely related to levels of childhood physical activity in different countries [22].

The Global Matrix 2.0 is a comprehensive summary of physical activity behavior and sources of influence indicators from 38 countries [18]. Their data suggest strengths and limitations across countries, with some global patterns emerging when comparing countries clustered by continent, human development index (HDI), and inequality. There is some evidence of higher physical activity and lower sedentary behavior in countries reporting poorer infrastructure and a greater reliance on Active Play and Active Transportation; and lower physical activity and higher sedentary behavior in countries reporting better infrastructure and a greater reliance on Organized Sport Participation and better School and Community facilities and policies. This paradox suggests autonomy to play and greater independent mobility rather than infrastructure and structured activities may facilitate higher levels of physical activity [18]. In our study, maternal education and number of people in the family (which can be considered as an income proxy) may represent possible social determinants; despite the high number of people with a high level of education in the sample, the difference between countries was relevant.

Another point to be discussed is the pronounced change of physical activity status in countries with high excessive weight rates, such as Brazil and Chile (22.2% [23] and 39.8% [5], respectively). Even though our study does not provide data on the nutritional status of the population, the relationship between physical inactivity and excessive weight is well known. Although regions in Europe, such as Spain and Italy, are severely affected by the Covid-19 pandemic and by the most restrictive measures of social isolation, the variation in the status of physical activity in these regions was not as strong as that observed in Latin America countries. This might be due to the population’s habit of performing more physical activity compared to countries with a worse economic situation [24]. A longitudinal observational study carried out in Italy with obesity children evaluated whether confinement could change lifestyle habits in this population. Considering physical activity, the time spent doing sports decreased significantly by 2.30 ± 4.60 h/week and, depending on the duration, the lockdown effect may reflect the level of adiposity in children and adolescents [25].

Mediouni et al. [22] reported the impact of depression on obesity, termed “depreobesity”. In lockdown, physical activity is reduced because of school closures. Consequently, stress and depression might lead to an increased consumption of unhealthy food, longer TV time, and irregular sleep patterns, which are risk factors for obesity [26]. During lockdowns, reduced energy expenditure also contributes to the development of obesity [22]. Chen et al. [27] suggested that regular physical activities may also help children and adolescents to recover from the stress and anxiety they usually experience during lockdowns. Until the discovery of more effective measures to control SARS-CoV-2, promoting physical exercises during lockdown can be a good strategy not only for weight control, but can also for improving mental health and for the immune system [22].

In terms of diet, previous research with children and adolescents have shown very similar results to the ones found in our study, considering the elevated contribution of ultra-processed foods in the adolescents’ diet. A longitudinal study of 345 Brazilian children found that the percentage of energy these group obtained from such foods was approximately 49% [28].

A study conducted with the objective of investigating regular consumption, i.e., ≥5 days/week, of four groups of ultra-processed foods among adolescents from the National School Health Survey found high proportions of adolescents who consumed sweets, soft drinks, sweet cookies, and sausages (50.9%, 37.2%, 33.6%, and 18%, respectively) [29]. Another study among schoolchildren in New Zealand showed that those who were more sedentary were more likely to have high consumption of foods commonly advertised on television, namely soft drinks and artificial juices, sweets, snacks, and fast food items.

There are two possible explanations for high consumption of foods in this situation. First, the greater practicality inherent to ultra-processed foods favors the consumption of this type of food, while adolescents naturally exercise less [29]. In addition, children and adolescents who have had a longer period of sedentary behavior are likely to be more exposed to advertisements for foods considered to be ultra-processed. In this study, we do not demonstrate this association. A systematic review among children and adolescents shows that sedentary behavior was inversely associated with the consumption of fruits and vegetables and, positively related to the consumption of high energy density snacks, fast foods, and high density drinks [30]. Many studies found maternal education to be associated with ultra-processed food consumption. A cross-sectional study aiming to investigate the association between sedentary behavior and consumption of ultra-processed foods in Brazilian adolescents using data from the 2015 PeNSE (Pesquisa Nacional de Saúde do Escolar) showed results similar to those observed in the current study. In particular, the prevalence of daily consumption of ultra-processed foods were higher in the highest quintiles of maternal education [31].

Mais et al. [32] found that a mother’s low educational level is associated with a two-fold increase in the risk for the child to consume ultra-processed foods. A study carried out in Sweden concluded that children whose parents had less education tended to consume cheaper and less healthy foods [33]. This relationship may suggest that mothers with less education may have more difficulty in having access to information to choose healthier food [34]. Fernandez-Alvira et al. [35] noted that the consumption of chips, fast foods, sugary drinks, sweets, and chocolates increased in children as their parents’ schooling decreased.

A recent study showed that the country of residence is strongly related to food intake during the COVID-19 pandemic and to the modification of dietary trends among adolescents in this period when compared to the habitual diet. On the other hand, the habitual consumption of ultra-processed foods may be strongly related to the country’s continent. This reinforces the notion that many factors, such as socioeconomic status, fat diets, religion, and traditions of each country may influence dietary trends [36].

One difficulty of our study is that measuring physical activity, especially in adolescents, is challenging. There is evidence that the data collected by questionnaires only partially agree with those from more direct methods, such as accelerometers. Although the continuous scores of the questionnaires do not always agree with the continuous scores from accelerometry, the categorization of individuals into groups of physical activity shows moderate to high agreement between the instruments, which supports the use of questionnaires in systems monitoring [37]. As additional limitations, we can consider that sports at school and outside school were not investigated separately, in addition to not having data from TV time and use of electronic devices. Missing information about the general diet and the usual possible bias should be considered when dealing with food frequency questionnaires. In addition, our survey was based on a convenience sample from different countries, which might not reflect the diverse behaviors of the entire population from those places. Moreover, questionnaires that are assessed through social media could be subject to selection bias and the assessment of “before” retrospectively in survey-based studies might also have some limitations.

The strengths of this study are that we collected data from two distinct geographic regions and the investigation of a population often neglected in terms of monitoring health conditions, i.e., adolescents. The possibility of comparing the habits of this group before and during social isolation due to the Covid-19 pandemic is another strength of this study.

## 5. Conclusions

In conclusion, we report the first evidence of physical activity habits’ modifications in adolescents during Covid-19 confinement in two countries in Europe and three countries in Latin America. Despite the different determinants of physical inactivity in the population in both continents, we observed a fairly high percentage of physical inactivity in the population before and during the Covid-19 pandemic, which was worsened during the lockdown measures. The change in physical activity status was greater in Latin American countries. In addition, we also observed a high frequency of ultra-processed foods consumption among adolescents, which was also worsened by the lockdown. Again, Latin American countries exhibited higher habitual ultra-processed food consumption. Such findings reinforce the importance of developing public health policies for this group, focusing on measures to encourage a healthy lifestyle (diet and exercise), especially during and after periods of social isolation.

## Figures and Tables

**Table 1:** General characteristics of the sample (by country and overall).

	Brazil (*n* = 115)	Chile (*n* = 170)	Colombia (*n* = 161)	Spain (*n* = 147)	Italy (*n* = 133)	Overall (*n* = 726)
Age group (years)						
10–15	67 (58.3%)	106 (62.4%)	49 (30.4%)	66 (44.9%)	44 (33.1%)	332 (45.7%)
16–19	48 (41.7%)	64 (37.6%)	112 (69.6%)	81 (55.1%)	89 (66.9%)	394 (54.3%)
Sex						
Female	65 (56.5%)	97 (57.1%)	91 (56.5%)	87 (59.2%)	93 (69.9%)	433 (59.6%)
Male	50 (43.5%)	72 (42.4%)	69 (42.9%)	60 (40.8%)	38 (28.6%)	289 (39.8%)
Maternal education						
Middle school or less	7 (6.1%)	20 (11.8%)	17 (10.6%)	21 (14.3%)	1 (0.8%)	66 (9.1%)
High school	13 (11.3%)	42 (24.7%)	104 (64.6%)	28 (19.0%)	44 (33.1%)	231 (31.8%)
College	91 (79.1%)	105 (61.8%)	35 (21.7%)	92 (62.6%)	81 (60.9%)	404 (55.6%)
Does not know	4 (3.5%)	3 (1.8%)	5 (3.1%)	6 (4.1%)	7 (5.3%)	25 (3.4%)
Number of residents at home						
1–3 people	48 (41.7%)	60 (35.3%)	31 (19.3%)	23 (15.6%)	23 (17.3%)	185 (25.5%)
4 or more people	67 (58.3%)	110 (63.5%)	130 (80.7%)	124 (83.7%)	110 (82.7%)	541 (74.1%)
Lives with the father						
Does not live	0 (0%)	63 (37.1%)	41 (25.5%)	24 (16.3%)	15 (11.3%)	143 (19.7%)
Lives	115 (100%)	107 (62.9%)	120 (74.5%)	123 (83.7%)	118 (88.7%)	583 (80.3%)
Lives with the mother						
Does not live	9 (7.8%)	7 (4.1%)	24 (14.9%)	4 (2.7%)	2 (1.5%)	46 (6.3%)
Lives	106 (92.2%)	163 (95.9%)	137 (85.1%)	143 (97.3%)	131 (98.5%)	680 (93.7%)
PA ^1^ before						
Inactive	47 (40.9%)	136 (80.0%)	117 (72.7%)	116 (78.9%)	114 (85.7%)	530 (73.0%)
Active	68 (59.1%)	34 (20.0%)	44 (27.3%)	31 (21.1%)	19 (14.3%)	196 (27.0%)
PA^1^ during						
Inactive	107 (93.0%)	154 (90.6%)	114 (70.8%)	104 (70.7%)	98 (73.7%)	577 (79.5%)
Active	8 (7.0%)	16 (9.4%)	47 (29.2%)	43 (29.3%)	35 (26.3%)	149 (20.5%)
PA status						
Active before/during	6 (5.2%)	7 (4.1%)	23 (14.3%)	15 (10.2%)	12 (9.0%)	63 (8.7%)
Active during	2 (1.7%)	9 (5.3%)	24 (14.9%)	28 (19.0%)	23 (17.3%)	86 (11.8%)
Not active before/during	45 (39.1%)	127 (74.7%)	93 (57.8%)	88 (59.9%)	91 (68.4%)	444 (61.2%)
Not active during	62 (53.9%)	27 (15.9%)	21 (13.0%)	16 (10.9%)	7 (5.3%)	133 (18.3%)
Change in PA status						
Status changed	64 (55.7%)	36 (21.2%)	45 (28.0%)	44 (29.9%)	30 (22.6%)	219 (30.2%)
Status did not change	51 (44.3%)	134 (78.8%)	116 (72.0%)	103 (70.1%)	103 (77.4%)	507 (69.8%)
Ultra-processed foods consumption						
≥5 ×/week	70 (60.9%)	118 (69.4%)	96 (59.6%)	115 (78.2%)	88 (66.1%)	487 (81.3%)
≤5 ×/week	45 (39.1%)	52 (22.6%)	65 (41.4%)	32 (21.8%)	45 (33.9%)	239 (18.7%)

^1^ PA: physical activity.

**Table 2:** General characteristics of the sample according to the physical activity status before and during the quarantine period.

	Active Before/During (*n* = 63)	Active During (*n* = 86)	Not Active Before/During (*n* = 444)	Not Active During (*n* = 133)	*p*-Value *
Country					
Brazil	6 (9.5%)	2 (2.3%)	45 (10.1%)	62 (46.6%)	<0.001
Chile	7 (11.1%)	9 (10.5%)	127 (28.6%)	27 (20.3%)	
Colombia	23 (36.5%)	24 (27.9%)	93 (20.9%)	21 (15.8%)	
Spain	15 (23.8%)	28 (32.6%)	88 (19.8%)	16 (12.0%)	
Italy	12 (19.0%)	23 (26.7%)	91 (20.5%)	7 (5.3%)	
Continent					
Europe	27 (42.9%)	51 (59.3%)	179 (40.3%)	23 (17.3%)	<0.001
Latin America	36 (57.1%)	35 (40.7%)	265 (59.7%)	110 (82.7%)	
Age group (years)					
10–15	25 (39.7%)	40 (46.5%)	204 (45.9%)	63 (47.4%)	0.777
16–19	38 (60.3%)	46 (53.5%)	240 (54.1%)	70 (52.6%)	
Sex					
Female	27 (42.9%)	54 (62.8%)	274 (61.7%)	78 (58.6%)	0.031
Male	36 (57.1%)	32 (37.2%)	167 (37.6%)	54 (40.6%)	
Maternal education					
Middle school or less	6 (9.5%)	14 (16.3%)	39 (8.8%)	7 (5.3%)	0.034
High school	24 (38.1%)	28 (32.6%)	145 (32.7%)	34 (25.6%)	
College	30 (47.6%)	42 (48.8%)	244 (55.0%)	88 (66.2%)	
Does not know	3 (4.8%)	2 (2.3%)	16 (3.6%)	4 (3.0%)	
Number of residents at home					
1–3 people	8 (12.7%)	16 (18.6%)	112 (25.2%)	49 (36.8%)	0.001
4 or more people	55 (87.3%)	70 (81.4%)	329 (74.1%)	84 (63.2%)	
Lives with the father					
Does not live	12 (19.0%)	20 (23.3%)	90 (20.3%)	21 (15.8%)	0.556
Lives	51 (81.0%)	66 (76.7%)	354 (79.7%)	112 (84.2%)	
Lives with the mother					
Does not live	3 (4.8%)	5 (5.8%)	28 (6.3%)	10 (7.5%)	0.893
Lives	60 (95.2%)	81 (94.2%)	416 (93.7%)	123 (92.5%)	

PA: physical activity. * *p*-values for the Chi-squared tests.

**Table 3:** Results of the multivariate regression for the physical activity status.

	Active Before/During *		Active During *		Inactive During *	
	OR (95% CI)	*p*-Value	OR (95% CI)	*p*-Value	OR (95% CI)	*p*-Value
Crude model						
Continent						
Latin America **	0.90 (0.53–1.54)	0.701	0.46 (0.29–0.74)	0.001	3.23 (1.98–5.26)	<0.001
Adjusted model						
Continent						
Latin America	0.85 (0.48–.51)	0.586	0.42 (0.26–0.70)	0.001	2.98 (1.80–4.94)	<0.001
Sex						
Male	2.22 (1.28–3.86)	0.005	1.04 (0.63–1.70)	0.878	0.96 (0.63–1.46)	0.858
Maternal education						
High school	0.99 (0.37–2.62)	0.982	0.53 (0.25–1.12)	0.096	1.35 (0.55–3.32)	0.507
College	0.69 (0.26–1.79)	0.445	0.41 (0.20–0.84)	0.015	2.32 (0.99–5.44)	0.053
Number of residents at home						
4 or more people	2.40 (1.05–5.53)	0.039	1.22 (0.67–2.24)	0.518	0.65 (0.42–1.01)	0.42

* Note: Reference category: Inactive before/during; ** Reference: Europe.

**Table 4:** Multiple logistic regression model of the variables associated with ultra-processed food consumption.

Variables	Crude Model			Adjusted Model		
	OR	IC95%	*p*	OR	IC95%	*P*
**Maternal Education**						
High School College	0.73 EU	0.45–1.21	0.130	0.76 EU	0.44–1.21	0.235
**Time of Physical Activity**						
<60 min/day ≥60 min/day	0.90 EU	0.66–1.23	0.123	0.80 EU	0.58–1.11	0.200
**Continent**						
Latin America Europe	1.50 EU	1.08–2.08	0.014	1.58 EU	1.13–2.22	0.007

CI: confidence interval. Controlled variables in the model: sex, age, and number of people at home. EU: European Union; ≥60 min/day; College.

## Supplementary Materials

The following are available online at https://www.mdpi.com/2072-6643/12/8/2289/s1, Table S1: Online survey used for the present study (in Spanish).
