# Supplementary material for: Changes of Physical Activity and Ultra-Processed Food Consumption in Adolescents from Different Countries during Covid-19 Pandemic: An Observational Study
Source: Nutrients. 2020 Jul 30;12(8):2289. doi: 10.3390/nu12082289 (PMC7468997; doi:10.3390/nu12082289)
Supplement: Supplementary file 1 [file nutrients-12-02289-s001.pdf]

Supplementary Table S1: Online survey used for the present study (in Spanish).

“Estudio observacional de la modificación del estilo de vida de adolescentes de diferentes países durante el confinamiento por Covid-19”

Se le invita a participar en un estudio de investigación denominado “Estudio observacional de la modificación del estilo de vida de adolescentes de diferentes países durante el confinamiento por Covid-19”.
El promotor del estudio, así como el instituto madrileño de Investigaciones Avanzadas en Alimentación (IMDEA Alimentación), le agradece se tome unos minutos para leer la hoja de información y participar en el estudio que se explica a continuación.
El objetivo general de IMDEA Alimentación es llevar a cabo una investigación de primer nivel en alimentación, nutrición y salud, e identificar estrategias nutricionales de valor económico y social. Desde IMDEA Alimentación queremos realizar un estudio observacional para poder describir el estilo de vida de los adolescentes de España, Brasil, Chile, Colombia e Italia durante el confinamiento por COVID-19, y los posibles cambios en sus hábitos dietéticos y de actividad física debidos a esta situación comparado con los observados antes del confinamiento. Estos datos nos ayudarán a poder promover propuestas para educar a la población en unos hábitos dietéticos adecuados y llevar un estilo de vida saludable. La nutrición puede ser una herramienta eficaz en la prevención y el tratamiento de enfermedades crónicas, ya que se ha demostrado que diferentes nutrientes y productos alimenticios desempeñan funciones vitales en los mecanismos moleculares del cuerpo.
Para la recogida de datos del estudio, tendrá que rellenar el presente formulario acerca de sus hábitos alimenticios y de actividad física, antes y durante el confinamiento por COVID-19.
La encuesta le llevará unos 20 minutos y será de gran ayuda al mundo científico y médico.

PROTECCIÓN DE DATOS PERSONALES
El promotor del estudio se compromete al cumplimiento de la Ley Orgánica 3/2018, de 5 de diciembre, de Protección de Datos Personales y garantía de los derechos digitales. Los datos recogidos para el estudio estarán identificados mediante un código, de manera que no incluya información que pueda identificarle. Por lo tanto, su identidad no será revelada a persona alguna salvo excepciones en caso de requerimiento legal. El tratamiento, la comunicación y la cesión de los datos de carácter personal de todos los participantes se ajustarán a lo dispuesto en esta ley.
El acceso a su información personal identificada quedará restringido a los colaboradores del estudio y al Comité de Ética de la Investigación, cuando lo precisen para comprobar los datos y procedimientos del estudio, pero siempre manteniendo la confidencialidad de los mismos de acuerdo a la legislación vigente.
De acuerdo a lo que establece la legislación de protección de datos, usted puede ejercer los derechos de acceso, modificación, oposición y cancelación de datos, para lo cual deberá dirigirse al promotor del estudio.
Si usted decide retirar el consentimiento para participar en este estudio, ningún dato será añadido a la base de datos. Los datos codificados pueden ser transmitidos a terceros y a otros países, pero en ningún caso contendrán información que le pueda identificar directamente, como nombre y apellidos, iniciales, dirección, nº de la seguridad social, etc. En el caso de que se produzca esta cesión, será para los mismos fines del estudio descrito o para su uso en publicaciones científicas, pero siempre manteniendo la confidencialidad de los mismos de acuerdo a la legislación vigente.

COMPENSACIÓN ECONÓMICA
La participación en el estudio no supondrá ningún gasto para usted ni recibirá compensación económica por ello.

CONSENTIMIENTO INFORMADO
Al aceptar y rellenar esta encuesta está dando su consentimiento para participar en dicho estudio, y declara haber leído la hoja de información, la veracidad de la información consignada, comprender que su participación es voluntaria y que puede eliminar sus datos del estudio cuando quiera y sin tener que dar explicaciones, escribiendo al correo alimentacion.imdea@gmail.com.

ESTA ENCUESTA ES ANÓNIMA Y TODOS LAS RESPUESTAS SE TRATARÁN DE FORMA CONFIDENCIAL.
*Obligatorio

1. ¿Acepta participar y declara haber leído la hoja de información, la veracidad de la información consignada, comprender que su participación es voluntaria y que puede eliminar sus datos del estudio cuando quiera y sin tener que dar explicaciones? *

Marca solo un óvalo.

- ◯ SI
- ◯ No

Cuestionario de hábitos de vida

2. ¿En qué país resides? *

Marca solo un óvalo.

- ◯ España
- ◯ Italia
- ◯ Colombia
- ◯ Chile
- ◯ Brasil

3. ¿En qué ciudad resides? *

____

4. ¿Cuántos años tienes? *

Marca solo un óvalo.

- 13
- 14
- 15
- 16
- 17
- 18
- 19

5. ¿Eres hombre o mujer? *

Marca solo un óvalo.

- Hombre
- Mujer
- Prefiero no decirlo

6. ¿En qué curso estás? *

Marca solo un óvalo.

- 5º de primaria
- 6º de primaria
- 1º de E.S.O.
- 2º de E.S.O.
- 3º de E.S.O.
- 4º de E.S.O.
- 1º de bachillerato
- 2º de bachillerato
- Estudiante universitario
- Formación profesional
- Otros

7. ¿Vives con tu padre? *

Marca solo un óvalo.

- Si
- No

8. ¿Vives con tu madre? *

Marca solo un óvalo.

- Sí
- No

9. Contando contigo, ¿cuántas personas viven tu casa? *

Marca solo un óvalo.

- 1 (Sólo tú)
- 2
- 3
- 4
- 5
- 6
- 7
- 8
- 9
- 10 o más

10. ¿Qué nivel de estudios tiene tu madre/padre? *

Marca solo un óvalo.

- Grado universitario
- Formación profesional
- Bachiller
- Estudios básicos
- Sin estudios
- Otros
- No sé

Cuestionario de alimentación

Las siguientes preguntas serán de tu alimentación ahora y antes del confinamiento en casa por el coronavirus.

11. ¿Estos últimos 7 días, has desayunado? *

Marca solo un óvalo.

- Sí
- No
- Sólo a veces

12. Antes del confinamiento en casa, ¿Desayunabas? *

Marca solo un óvalo.

- Sí
- No
- A veces

13. En los últimos 7 días ¿Has almorzado o cenado con tu madre o padre? *

Marca solo un óvalo.

- Sí
- No
- A veces

14. Antes del confinamiento, ¿Almorzabas o cenabas con tus padres? *

Marca solo un óvalo.

- Sí
- No
- A veces

15. En los últimos 7 días, ¿Sueles comer viendo la televisión o estudiando? *

Marca solo un óvalo.

- Si
- No
- A veces

16. Antes del confinamiento, ¿ Comías viendo la televisión o estudiando? *

Marca solo un óvalo.

- Si
- No
- A veces

17. En los últimos 7 días, ¿Cuántos días has comido legumbres (garbanzos, lentejas, judías...)? *

Marca solo un óvalo.

- Todos los días
- 6 días en los últimos 7 días
- 5 días en los últimos 7 días
- 4 días en los últimos 7 días
- 3 días en los últimos 7 días
- 2 días en los últimos 7 días
- Un día en los últimos 7 días
- Ningún día en los últimos 7 días

18. Y antes del confinamiento, ¿Cuántos días a la semana solías comer legumbres (garbanzos, lentejas, judías...)? *

Marca solo un óvalo.

- Todos los días de la semana
- 6 días a la semana
- 5 días a la semana
- 4 días a la semana
- 3 días a la semana
- 2 días a la semana
- Un día a la semana
- Menos de un día a la semana
- Nunca solía comer legumbres

19. En los últimos 7 días, ¿Cuántos días has comido fritos (patatas fritas, nuggets, palitos de pescado fritos, refrigerados fritos...)? *

Marca solo un óvalo.

- Todos los días
- 6 días en los últimos 7 días
- 5 días en los últimos 7 días
- 4 días en los últimos 7 días
- 3 días en los últimos 7 días
- 2 días en los últimos 7 días
- Un día en los últimos 7 días
- Ningún día en los últimos 7 días

20. Y antes del confinamiento, ¿cuántos días comías fritos (patatas fritas, nuggets, palitos de pescado fritos, refrigerados fritos...) a la semana? *

Marca solo un óvalo.

- Todos los días a la semana
- 6 días a la semana
- 5 días a la semana
- 4 días a la semana
- 3 días a la semana
- 2 días a la semana
- Un día a la semana
- Menos de un día a la semana
- Nunca solía comer fritos

21. En los últimos 7 días, ¿Cuántos días has comido al menos un tipo de verdura? Ejemplos: lechuga, calabaza, brócoli, cebolla, zanahoria, col rizada, espinaca, pepino, pimiento, etc. No incluye patatas ni yuca. *

Marca solo un óvalo.

- Todos los días
- 6 días en los últimos 7 días
- 5 días en los últimos 7 días
- 4 días en los últimos 7 días
- 3 días en los últimos 7 días
- 2 días en los últimos 7 días
- Un día en los últimos 7 días
- Ningún día en los últimos 7 días

22. Y antes del confinamiento, ¿Cuántos días comías al menos un tipo de verdura a la semana? Ejemplos: lechuga, calabaza, brócoli, cebolla, zanahoria, col rizada, espinaca, pepino, pimiento, etc. No incluye patatas ni yuca. *

Marca solo un óvalo.

- Todos los días a la semana
- 6 días a la semana
- 5 días a la semana
- 4 días a la semana
- 3 días a la semana
- 2 días a la semana
- Un día a la semana
- Menos de un día a la semana
- Nunca solía comer verduras

23. En los últimos 7 días, ¿cuántos días has comido golosinas (dulces, dulces, chocolates, chicles, chocolates o piruletas)? *

Marca solo un óvalo.

- Todos los días
- 6 días en los últimos 7 días
- 5 días en los últimos 7 días
- 4 días en los últimos 7 días
- 3 días en los últimos 7 días
- 2 días en los últimos 7 días
- Un día en los últimos 7 días
- Ningún día en los últimos 7 días

24. Y antes del confinamiento, ¿Cuántos días comías golosinas (dulces, dulces, chocolates, chicles, chocolates o piruletas) a la semana? *

Marca solo un óvalo.

- Todos los días a la semana
- 6 días a la semana
- 5 días a la semana
- 4 días a la semana
- 3 días a la semana
- 2 días a la semana
- Un día a la semana
- Menos de un día a la semana
- Nunca solía comer golosinas

25. En los últimos 7 días, ¿cuántos días has comido fruta fresca o ensalada de frutas? *

Marca solo un óvalo.

- Todos los días
- 6 días en los últimos 7 días
- 5 días en los últimos 7 días
- 4 días en los últimos 7 días
- 3 días en los últimos 7 días
- 2 días en los últimos 7 días
- Un día en los últimos 7 días
- Ningún día en los últimos 7 días

26. Y antes del confinamiento, ¿Cuántos días comías fruta fresca o ensalada de frutas a la semana? *

Marca solo un óvalo.

- Todos los días a la semana
- 6 días a la semana
- 5 días a la semana
- 4 días a la semana
- 3 días a la semana
- 2 días a la semana
- Un día a la semana
- Menos de un día a la semana
- Nunca solía comer fruta

27. En los últimos 7 días, ¿cuántos días has bebido bebidas azucaradas o refrescos (coca-cola, fanta, nestea...) ? *

Marca solo un óvalo.

- Todos los días
- 6 días en los últimos 7 días
- 5 días en los últimos 7 días
- 4 días en los últimos 7 días
- 3 días en los últimos 7 días
- 2 días en los últimos 7 días
- Un día en los últimos 7 días
- Ningún día en los últimos 7 días

28. Y antes del confinamiento, ¿cuántos días bebías bebidas azucaradas o refrescos a la semana? *

Marca solo un óvalo.

- Todos los días a la semana
- 6 días a la semana
- 5 días a la semana
- 4 días a la semana
- 3 días a la semana
- 2 días a la semana
- Un día a la semana
- Menos de un día a la semana
- Nunca solía beber refrescos

29. En los últimos 7 días, ¿Cuántos días has comido alimentos ultraprocesados, como hamburguesa, jamón, mortadela, salami, salchicha, salchicha, fideos instantáneos, aperitivos envasados, galletas saladas, alimentos precocinados? *

Marca solo un óvalo.

- Todos los días
- 6 días en los últimos 7 días
- 5 días en los últimos 7 días
- 4 días en los últimos 7 días
- 3 días en los últimos 7 días
- 2 días en los últimos 7 días
- Un día en los últimos 7 días
- Ningún día en los últimos 7 días

30. Y antes del confinamiento, ¿Cuántos días comías alimentos ultraprocesados, como hamburguesa, jamón, mortadela, salami, salchicha, salchicha, fideos instantáneos, aperitivos envasados, galletas saladas, alimentos precocinados a la semana? *

Marca solo un óvalo.

- Todos los días a la semana
- 6 días a la semana
- 5 días a la semana
- 4 días a la semana
- 3 días a la semana
- 2 días a la semana
- Un día a la semana
- Menos de un día a la semana
- Nunca solía comer alimentos de este tipo

31. En los últimos 7 días, ¿Cuántos días has pedido comida de restaurantes de comida rápida como bares, puestos de perritos calientes, pizzerías, restaurantes, chino, etc.? *

Marca solo un óvalo.

- Todos los días
- 6 días en los últimos 7 días
- 5 días en los últimos 7 días
- 4 días en los últimos 7 días
- 3 días en los últimos 7 días
- 2 días en los últimos 7 días
- Un día en los últimos 7 días
- Ningún día en los últimos 7 días

32. Y antes del confinamiento, ¿Cuántos días a la semana consumías comida de restaurantes de comida rápida como bares, puestos de perritos calientes, pizzerías, restaurantes, etc.? *

Marca solo un óvalo.

- Todos los días a la semana
- 6 días a la semana
- 5 días a la semana
- 4 días a la semana
- 3 días a la semana
- 2 días a la semana
- Un día a la semana
- Menos de un día a la semana
- Nunca solía comer en restaurantes ni comida rápida

En los últimos 30 días...

Ahora contesta 4 preguntas sobre lo que has comido durante los últimos 30 días, en este período de cuarentena o confinamiento por el coronavirus.

33. En los últimos 30 días, ¿Cuántas veces al día comes normalmente fruta fresca o ensalada de frutas? *

Marca solo un óvalo.

- 5 veces al día o más
- 4 veces al día
- 3 veces al día
- 2 veces al día
- Una vez al día
- Menos de una vez al día en los últimos 30 días
- Nunca, en los últimos 30 días

34. En los últimos 30 días, ¿Cuántas veces al día has comido verduras como lechuga, calabaza, brócoli, cebolla, zanahoria, col rizada, espinacas, pepino, tomate, etc.? No incluye patatas y yuca. *

Marca solo un óvalo.

- 5 veces al día o más
- 4 veces al día
- 3 veces al día
- 2 veces al día
- Una vez al día
- Menos de una vez al día en los últimos 30 días
- Nunca, en los últimos 30 días

35. En los últimos 30 días, ¿Cuántas veces al día has bebido bebidas azucaradas? *

Marca solo un óvalo.

- 5 veces al día o más
- 4 veces al día
- 3 veces al día
- 2 veces al día
- Una vez al día
- Menos de una vez al día en los últimos 30 días
- No he bebido refrescos ni bebidas azucaradas en los últimos 30 días

36. En los últimos 30 días, ¿tú o alguien de su familia ha pedido comida de restaurantes, pizzeria, comida rápida, chino, etc...? *

Marca solo un óvalo.

- Si, 5 veces a la semana o más
- Si, 4 veces a la semana
- Si, 3 veces a la semana
- Si, 2 veces a la semana
- Si, una vez a la semana
- Menos de una vez a la semana
- No hemos pedido comida de restaurantes, pizzerías, etc. en los últimos 30 días

Cuestionario de actividad física

Ya estamos acabando!! Las siguientes preguntas serán de tu actividad física en la última semana y antes del confinamiento.

37. Durante los últimos 7 días, ¿Cuántos días has realizado actividades físicas vigorosas como levantar objetos pesados, excavar, aeróbicos, o pedalear rápido en bicicleta? *

Marca solo un óvalo.

- Todos los días
- 6 días en los últimos 7 días
- 5 días en los últimos 7 días
- 4 días en los últimos 7 días
- 3 días en los últimos 7 días
- 2 días en los últimos 7 días
- Un día en los últimos 7 días
- No he realizado ninguna actividad física vigorosa

38. En los últimos 7 días, ¿Cuánto tiempo estuviste haciendo la actividad física vigorosa en uno de esos días que las realizaste? (levantar objetos pesados, excavar, aeróbicos, o pedalear rápido en bicicleta) *

Marca solo un óvalo.

- 8 horas o más
- 7 horas
- 6 horas
- 5 horas
- 4 horas
- 3 horas
- 2 horas
- Una hora
- Media hora
- Menos de media hora
- No he realizado ninguna actividad física vigorosa

39. Y antes del confinamiento, ¿Cuántos días a la semana realizabas actividades físicas vigorosas como levantar objetos pesados, excavar, aeróbicos, o pedalear rápido en bicicleta? *

Marca solo un óvalo.

- Todos los días a la semana
- 6 días a la semana
- 5 días a la semana
- 4 días a la semana
- 3 días a la semana
- 2 días a la semana
- Un día a la semana
- No he realizaba ninguna actividad física vigorosa antes del confinamiento

40. Y antes del confinamiento ¿Cuánto tiempo usualmente estuviste haciendo la actividad física vigorosa en uno de esos días en que las realizaste? (levantar objetos pesados, excavar, aeróbicos, o pedalear rápido en bicicleta). *

Marca solo un óvalo.

- 8 horas o más
- 7 horas
- 6 horas
- 5 horas
- 4 horas
- 3 horas
- 2 horas
- Una hora
- Media hora
- Menos de media hora
- No he realizaba ninguna actividad física vigorosa antes del confinamiento

41. Durante los últimos 7 días, ¿Cuántos días has realizado actividades físicas moderadas como cargar objetos livianos, pedalear en bicicleta a paso regular, o jugar dobles de tenis? *

Marca solo un óvalo.

- Todos los días
- 6 días en los últimos 7 días
- 5 días en los últimos 7 días
- 4 días en los últimos 7 días
- 3 días en los últimos 7 días
- 2 días en los últimos 7 días
- Un día en los últimos 7 días
- No he realizado ninguna actividad física moderada

42. En los últimos 7 días, ¿Cuánto tiempo estuviste haciendo la actividad física moderada en uno de esos días en que la realizaste? (cargar objetos livianos, pedalear en bicicleta a paso regular, o jugar dobles de tenis) *

Marca solo un óvalo.

- 8 horas o más
- 7 horas
- 6 horas
- 5 horas
- 4 horas
- 3 horas
- 2 horas
- Una hora
- Media hora
- Menos de media hora
- No he realizado ninguna actividad física moderada

43. Y antes del confinamiento, ¿Cuántos días a la semana realizabas actividades físicas moderadas como cargar objetos livianos, pedalear en bicicleta a paso regular, o jugar dobles de tenis? *

Marca solo un óvalo.

- Todos los días a la semana
- 6 días a la semana
- 5 días a la semana
- 4 días a la semana
- 3 días a la semana
- 2 días a la semana
- Un día a la semana
- No he realizaba ninguna actividad física moderada antes del confinamiento

44. Y antes del confinamiento ¿Cuanto tiempo usualmente hacías actividad física moderada en uno de esos días que la realizaste? (Cargar objetos livianos, pedalear en bicicleta a paso regular, o jugar dobles de tenis) *

Marca solo un óvalo.

- 8 horas o más
- 7 horas
- 6 horas
- 5 horas
- 4 horas
- 3 horas
- 2 horas
- Una hora
- Media hora
- Menos de media hora
- No he realizaba ninguna actividad física moderada antes del confinamiento

45. Durante los últimos 7 días, ¿Cuántos días has caminado al menos 10 minutos seguidos? *

Marca solo un óvalo.

- Todos los días
- 6 días en los últimos 7 días
- 5 días en los últimos 7 días
- 4 días en los últimos 7 días
- 3 días en los últimos 7 días
- 2 días en los últimos 7 días
- Un día en los últimos 7 días
- No he caminado en los últimos 7 días más de 10 minutos seguidos

46. Y en los últimos 7 días, ¿Cuanto tiempo estuviste caminado en uno de esos días que pudiste caminar? *

Marca solo un óvalo.

- 8 horas o más
- 7 horas
- 6 horas
- 5 horas
- 4 horas
- 3 horas
- 2 horas
- Una hora
- Media hora
- Menos de media hora
- No he caminado en los últimos 7 días

47. Y antes del confinamiento, ¿Cuántos días a la semana caminabas al menos 10 minutos? *

Marca solo un óvalo.

- Todos los días a la semana
- 6 días a la semana
- 5 días a la semana
- 4 días a la semana
- 3 días a la semana
- 2 días a la semana
- Un día a la semana
- No caminaba más de 10 minutos nunca

48. Y antes del confinamiento ¿Cuanto tiempo usualmente caminabas en uno de esos días que salías a caminar? *

Marca solo un óvalo.

- 8 horas o más
- 7 horas
- 6 horas
- 5 horas
- 4 horas
- 3 horas
- 2 horas
- Una hora
- Media hora
- Menos de media hora
- No caminaba más de 10 minutos nunca

49. Y en los últimos 7 días, ¿Cuanto tiempo has permanecido sentado al día? *

Marca solo un óvalo.

- 8 horas o más
- 7 horas
- 6 horas
- 5 horas
- 4 horas
- 3 horas
- 2 horas
- Una hora
- Media hora
- Menos de media hora
- No he estado sentado en los últimos 7 días

50. Y antes del confinamiento, ¿Cuanto tiempo permanecías sentado al día? *

Marca solo un óvalo.

- 8 horas o más
- 7 horas
- 6 horas
- 5 horas
- 4 horas
- 3 horas
- 2 horas
- Una hora
- Media hora
- Menos de media hora
- No he estado sentado en los últimos 7 días

Este contenido no ha sido creado ni aprobado por Google.

Google Formularios
